# Supplementary material for: The potassium channel K2P2.1 shapes the morphology and function of brain endothelial cells via actin network remodeling
Source: Nat Commun. 2025 Jul 18;16:6622. doi: 10.1038/s41467-025-61816-9 (PMC12274505; doi:10.1038/s41467-025-61816-9)
Supplement: Supplementary file 1 — Supplementary Information [file 41467_2025_61816_MOESM1_ESM.pdf]

Supplementary Materials for

The potassium channel K2P2.1 shapes the morphology and function of brain endothelial cells via actin network remodeling

Stefanie Lichtenberg[1,2,†], Laura Vinnenberg[1,†], Falk Steffen[3], Isabelle Plegge[4], Nicholas Hanuscheck[3], Vera Dobelmann[1], Joel Gruchot[1], Christina B. Schroeter[1], Haribaskar Ramachandran[5], Beatrice Wasser[3], Derya Bachir[1], Christopher Nelke[1], Jonas Franz[6,7], Christoph Riethmüller[7], Stefan Tenzer[8], Ute Distler[8], Christina Francisca Vogelaar[3], Kristina Kusche-Vihrog[9], Boris Skryabin[10], Timofey S. Rozhdestvensky[10], Albrecht Schwab[11], Jean Krutmann[5], Andrea Rossi[5], Thomas Budde[12], Stefan Bittner[3], Sven G. Meuth[1,†], Tobias Ruck[1, 13†*]

*Corresponding author. Email: tobias.ruck@bergmannsheil.de

The PDF file includes:

Figs. S1 to S3
Table S1
Table S2
Supplementary Methods

Other Supplementary Materials for this manuscript include the following:

Movie S1

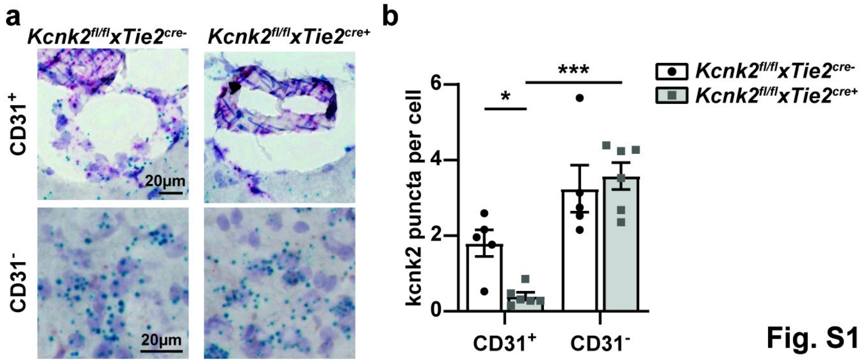

Fig. S1

Fig. S1. *Kcnk2* expression in brain endothelial cells of *Kcnk2$^{fl/fl}$xTie2^{cre+}* and *Kcnk2$^{fl/fl}$xTie2^{cre-}* mice. (**a**) BaseScope analysis of the exon 4 floxed region of *Kcnk2* demonstrates markedly diminished signal in CD31^{+} endothelial cells from Tie2^{cre+} animals while signal in non-endothelial cells is retained. Scale bars represent 20 µM. (**b**) Quantification of images shown in (a): *kcnk2* puncta per cell in CD31^{+} and CD31^{-} cells in endothelial cell-specific *Kcnk2$^{-/-}$* (*Kcnk2$^{fl/fl}$xTie2^{cre+}*; grey, grey cubes*)* and control (*Kcnk2$^{fl/fl}$xTie2^{cre-};* white, black dots*)* mice (N=5-6). N representing the number of individual mice. . Exact N-numbers for each condition are listed in the Source Data file. All data are shown as mean ±SEM. Statistical analysis using (b) 2-way ANOVA + Bonferroni correction with *p<0.05, ***p<0.001. Exact p-values are listed in the Source Data file.

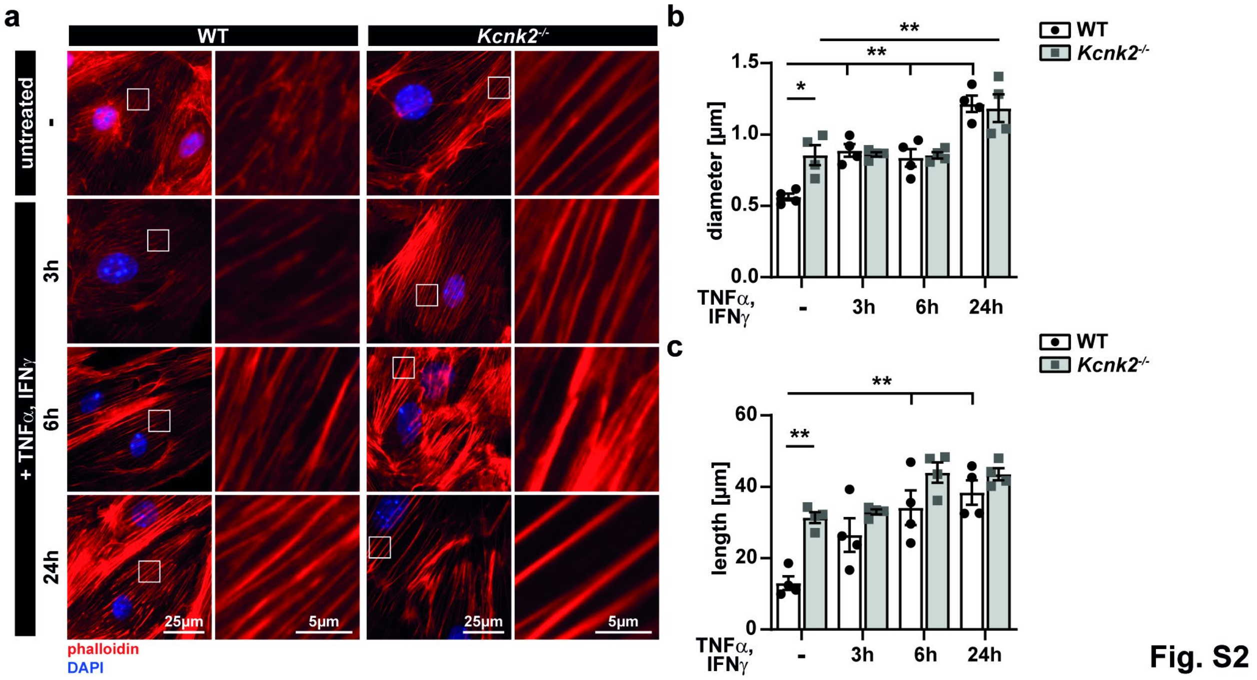

Fig. S2. Characterization of actin stress fibers in *Kcnk2*$^{-/-}$ and WT MBMECs. (a) Representative immunofluorescence images of untreated and TNFα, IFNγ treated (for 3 h, 6 h, 24 h) *Kcnk2*$^{-/-}$ and WT MBMECs. Actin is stained in red using phalloidin, nucleus in blue (DAPI). White boxes indicate the magnified image depicted on the right side of each panel. Scale bars represent 25 µm and 5 µm, respectively. (b) Measurement of actin fiber diameter of of WT (white, black dots) and *Kcnk2*$^{-/-}$ (grey, grey cubes) MBMECs in images from (a; N=4). (c) Quantification of average stress fiber length of images in (a; N=4). N representing the number of individual MBMEC preparations. All data are shown as mean ±SEM. Statistical analysis using (b, c) 1-way ANOVA + Bonferroni correction with *$p<0.05$ and **$p<0.01$. Exact p-values are listed in the Source Data file.

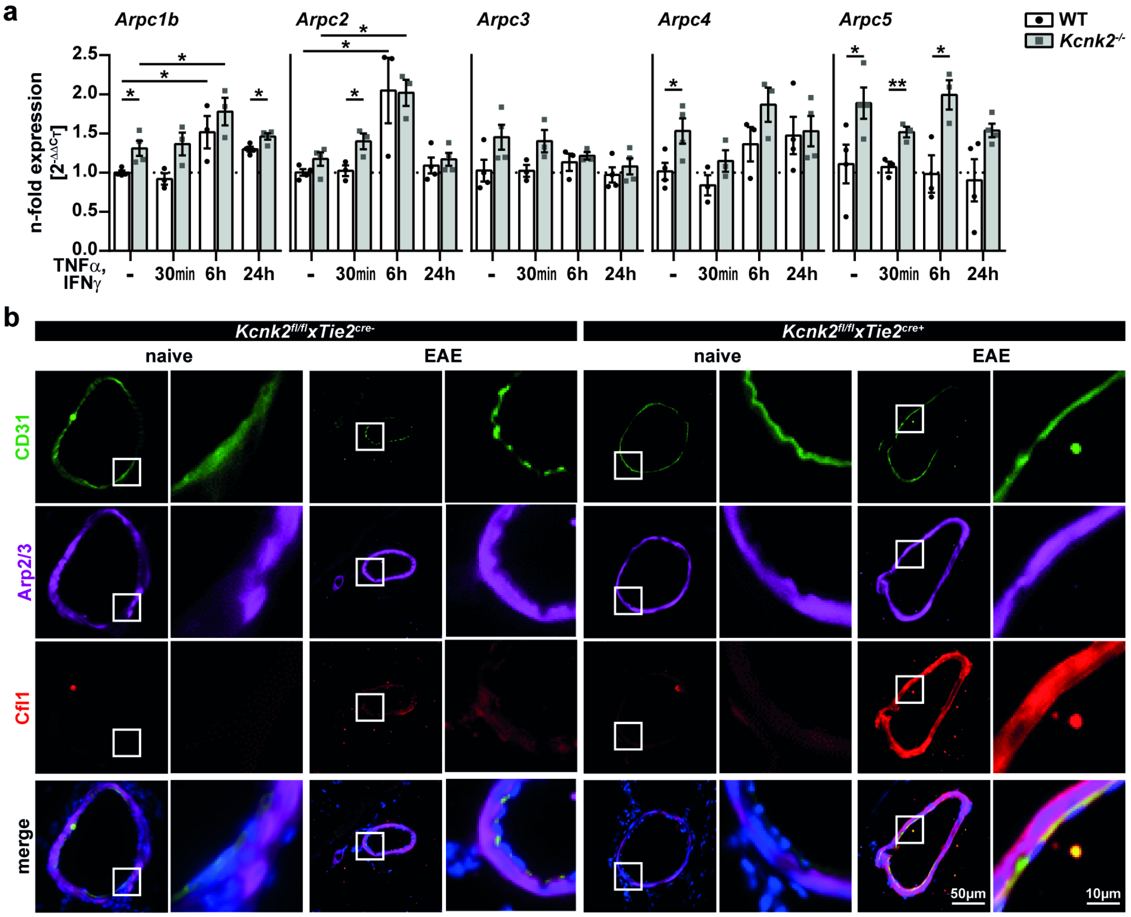

Fig. S3

Fig. S3. Charaterization of actin regulatory factors. (a) mRNA expression levels of *Arpc1b*, *Arpc2*, *Arpc3*, *Arpc4* and *Arpc5* in untreated and TNFα, IFNγ treated (for 3 h, 6 h, 24 h) WT (white, black dots) and *Kcnk2*$^{-/-}$ MBMECs (grey, grey cubes) assessed by qRT-PCR. Data are normalized to the respective untreated WT MBMECs control condition. Statistics were calculated with ΔC_T values (N=3-4). N representing the number of individual MBMEC preparations. Exact N-numbers for each condition are listed in the Source Data file. All data are shown as mean +/- SEM. Statistical analysis using 1-way ANOVA + Bonferroni correction with $^*p<0.05$ and $^{**}p<0.01$. Exact p-values are listed in the Source Data file. **(b)** Representative stainings of brain slices from naïve and EAE Kcnk2$^{fl/fl}$Tie2^{Cre+} and Kcnk2$^{fl/fl}$Tie2^{Cre-} animals. CD31 is stained in (green), Arp2/3 in magenta, Cfl1 in red and nuclei in blue (DAPI). White boxes indicate the magnified image depicted on the right side of each panel. Scale bars represent 50 µm and 10 µm, respectively.

Table S1.

Expression values of cytoskeletal regulators. Differentially regulated genes, determined by RT^2 qRT-PCR array. Fold changes (WT untreated as control group) of significantly regulated genes ($p < 0.05$; fold change cut off 1.25) are shown within the respective columns; unregulated genes are listed below (N=4). Statistical analysis using 1-way ANOVA + Bonferroni correction.

Gene name	*Kcnk2*$^{-/-}$ (control) [n-fold]	WT (inflamed) [n-fold]	*Kcnk2*$^{-/-}$ (inflamed) [n-fold]
Actr2			1.45
Actr3		1.75	1.60
Arfip2		-1.34	
Arhgdib		1.65	1.77
Arhgef11			-1.32
Arpc1b		1.29	
Arpc1b			1.45
Arpc4	1.50		1.49
Arpc5	1.85		2.09
Baiap2	1.67		
Cald1		2.10	
Calm1		-1.27	-1.43
Cdc42ep2		3.30	3.06
Cdk5		-2.06	-2.23
Cfl1	1.91	1.77	1.95
Clasp2			-1.26
Clip1			-1.51
Cyfip1	1.30		
Cyfip2	-2.06		
Dstn		2.39	2.10
Fnbp1l		-2.89	-2.33
Gsn		-3.16	-2-69
Iqgap1	-1.33	-1.32	
Iqgap2		-1.75	
Limk1		1.94	1.87
Limk2		-1.43	-1.71
Macf1			-1.76
Map3k11		-2.37	-3.01
Mapk13		6.62	5.85
Mapre1		1.43	-1.69
Mid1	1.55		1.62
Mylk		-2.24	

Mylk2	1.53		-1.50
Nck1		-1.43	-1.53
Nck2		-1.96	-2.85
Pak1		3.76	4.26
Pak4	-1.41	1.57	
Phldb2		-1.92	-1.90
Pikfyve		2.04	1.90
Ppp3ca		-1.54	-2.01
Racgap1			1.92
Rock1		1.40	1.59
Ssh1	-1.72	-1.40	
WasI		-1.78	-1.62
**not significantly regulated genes:** *Arap1, Arhgap6, Arpc2, Arpc3, Aurka, Aurkc, Cask, Ccna1, Ccnb2, Cdc42, Cdc42bpa, Cdc42ep3, Cdk5r1, Cit, Clasp1, Clip1, Clip2, Crk, Cttn, Diap1, Ezr, Fscn2, Llgl1, Mapre2, Mapt, Mark2, Msn, Map4, Pfn2, Ppp1r12a, Ppp1r12b, Ppp3cb, Rac1, Rdx, Rhoa, Ssh2, Stmn1, Tiam1, Vasp, Was, Wasf1*			

Table S2.

Extended statistical analysis. Summary of numbers of replicates and statistical methods in each experiment. "N" and "n" indicates the number of biological replicates per genotype and condition on which the statistical evaluation is based. If not stated otherwise 10 mice were pooled per genotype and MBMEC preparation, used for one specific experiment only and not shared across different assays.

Figure manuscript	Biological replicates	Further experimental details	Statistical method
1a	scheme		
1b	5-7 mice (N)	3 EAEs	representative images
1c	5-7 mice (N)	3 EAEs	representative images
1d	5-7 mice (N)	3 EAEs	representative images
1e	5-7 mice (N)	3 EAEs;<br>5 mice baseline,<br>7 mice isotype + spadin,<br>6 mice anti-ICAM1 + spadin	1way ANOVA, Bonferroni corr.
1f	8 mice (N)	3 EAEs	2-way ANOVA, Bonferroni corr.
1g	8 mice (N)	3 EAEs	2-way ANOVA, Bonferroni corr.
2a	4-6 MBMEC preparations (N)	2-3 technical replicates	Kruskal-Wallis test, Dunn`s multiple comparison test
2b	6 MBMEC preparations (N)		Kruskal-Wallis test, Dunn`s multiple comparison test
2c	scheme		
2d	16 MBMEC preparations (N)	12-16 analyzable flow chambers per condition; one T cell preparation per MBMEC preparation	1way ANOVA, Bonferroni corr.
3a	3 MBMEC preparations (N)	2 coverslips, 4 ROIs	representative images
3b	3 MBMEC preparations (N)	2 coverslips, 4 ROIs	representative images
3c	3 MBMEC preparations (N)	4-8 dishes	representative images
3d	3 MBMEC preparations (N)	4-8 dishes, 27-34 ROIs	1way ANOVA, Bonferroni corr.
3e	17-19 individual T cells (n)	≥17 T cell isolations (individual mice);<br>4-8 MBMEC preparations (N), 2 dishes per preparation;<br>20-30 endothelial cells with minimum 20 force-distance curves as technical replicates	Kruskal-Wallis test, Dunn`s multiple comparison test
4a	5 MBMEC preparations (N)	4 mice pooled	Student's t-test, Benjamini-Hochberg correction for multiple testing, FDR 0.01
4b	5 MBMEC preparations (N)	4 mice pooled	
4c	5 MBMEC preparations (N)	4 mice pooled	
4d	3 MBMEC preparations (N)	4-8 dishes<br>(12-22 ROIs)	representative images
4e	3 MBMEC preparations (N)	4-8 dishes, 12-22 ROIs	1way ANOVA, Bonferroni corr.

4f	45-59 single cells (n)	4 MBMEC preparations (N); 6 force distance curves per cell as technical replicates	1way ANOVA, Bonferroni corr.
5a	4 MBMEC preparations (N)	2-3 technical replicates	Significantly differentially regulated genes; 1way ANOVA, Bonferroni corr.
5b	4 MBMEC preparations (N)	2-3 technical replicates	1way ANOVA, Bonferroni corr.
5c	4 MBMEC preparations (N)	2-3 technical replicates	1way ANOVA, Bonferroni corr.
5d	4 MBMEC preparations (N)	2-3 technical replicates	1way ANOVA, Bonferroni corr.
5e	4 MBMEC preparations (N)	2 coverslips, 4 ROIs per coverslip	representative images
5f	4 MBMEC preparations (N)	2 coverslips, 4 ROIs per coverslip	1way ANOVA, Bonferroni corr.
6a	4 MBMEC preparations (N)	2 coverslips, 4 ROIs per coverslip	representative images
6b	4 MBMEC preparations (N)	2 coverslips, 4 ROIs per coverslip	1way ANOVA, Bonferroni corr.
6c	4 MBMEC preparations (N)	2 coverslips, 4 ROIs per coverslip	1way ANOVA, Bonferroni corr.
6d	4 MBMEC preparations (N)	duplicates, 4 ROIs each	representative images
6e	4 MBMEC preparations (N)	duplicates, 4 ROIs each	1way ANOVA, Bonferroni corr.
6f	5 MBMEC preparations (N)	quadruples, 4 ROIs each	representative images
6g	5 MBMEC preparations (N)	quadruples, 4 ROIs each	1way ANOVA, Bonferroni corr.
7a	12 MBMEC preparations (N)	2 coverslips, 4 ROIs per coverslip	representative images
7b	3 MBMEC preparations (N)	2-3 technical replicates	2-way ANOVA, Bonferroni corr.
7c	12 MBMEC preparations (N)	8-10 analyzable flow chambers per condition; one T cell preparation per MBMEC preparation	2-way ANOVA, Bonferroni corr.
8	scheme		
S1a	5-6 mice (N)	3 EAEs	representative images
S1b	5-6 mice (N)	3 EAEs, 2 ROIs per mouse	2-way ANOVA, Bonferroni corr.
S2a	4 MBMEC preparations (N)	2 coverslips, 4 ROIs per coverslip	representative images
S2b	4 MBMEC preparations (N)	2 coverslips, 4 ROIs per coverslip	1way ANOVA, Bonferroni corr.
S2c	4 MBMEC preparations (N)	2 coverslips, 4 ROIs per coverslip	1way ANOVA, Bonferroni corr.
S3a	3-4 MBMEC preparations (N)	quadruples, 2-3 technical replicates	1way ANOVA, Bonferroni corr.
S3b	5-6 mice (N)	3 EAEs, 2 ROIs per mouse	representative images
S-Tab 1	4 MBMEC preparations (N)	2-3 technical replicates	Significantly differentially regulated genes, 1way ANOVA Bonferroni corr.

Movie S1.

Supplementary Video 1. Intravital two-photon microscope imaging of brainstem of mice. Representative video of 2PM imaging of the brainstem after EAE induction, at a disease score of 2. Blood vessels were visualized by rhodamine-labelled dextran (red); Th17 T cells are tagged with GFP (green). Baseline recording was performed for 30 min, followed by spadin treatment for $K_{2P}2.1$ inhibition.

SUPPLEMENTARY METHODS

<u>Generation of *Kcnk2$^{fl/fl}$* (*Kcnk2_floxed*) mice</u>

Kcnk2_floxed mouse line was generated by direct oocytes microinjections using the CRISPRCas9 components together with the donor DNA template (*Kcnk2_templ*) with the subsequent chirurgical embryo transfer. *Kcnk2$^{fl/fl}$* mice were kept homozygous and backcrossed to C57Bl/6J background. Donor DNA template "*Kcnk2_templ*" was chemically synthesized [Biomatik, USA]. Mouse genomic sequences in *Kcnk2_templ* are labeled with the capital letters, the artificially inserted sequences are labeled with small letters:

TTAGAATAATTGCTTACATTAGCTTTTACTTGTACTGTTATAATATAAATTGAGTC
TTCCtgcagaagctgggccctggtaatataacttcgtatagcatacattatacgaagttatggctgctgcccagcaggaaacaaccta
cttgagggcTCTTGTAATAGCTGGTGATTTCTGGTTAAACTTGGCATGGGGTGTTTAAA
ACTATGATACATTTGTAAATAACTGGATTTTTCCATTTTTGGTTTTCTTTTTAGGAT
TTGGAAACATCTCCCCACGAACTGAAGGTGGAAAAATATTCTGCATCATCTATGC
CTTGCTGGGAATTCCCCTCTTTGGCTTTCTACTGGCTGGGGTTGGTGATCAGCTAG
GAACTATATTTGGAAAAGGAATTGCCAAAGTGGAAGACACATTTATTGTGAGTAG
CACAAACTTCTTGCTACATCTATTTAATGGTTTTGAAAATATGTTACATATTCTAG
CACCTTAGATAATGTAGAggatccaggttaggatcgcataggataacttcgtatagcatacattatacgaagttatggct
gctgcccagcatccttaatgcgcgtagtcgAGCTTGCCACTAACTGTGTTTGACTTATTTACATACT
TGATTGAAAGAAACTGAAAATTA.

<u>Mouse oocytes microinjections:</u> For the preparation of CRISPR-Cas9 microinjection solution, commercially synthesized crRNA's: Kcnk2_crR12 with target sequence: CTGGATTGTTTATTTCACCT, and Kcnk2_crR16

(ACAGTTAGTGGCAAGCTAAG), together with the tracrRNA and, Cas9 protein [Integrated DNA Technologies (IDT), USA] were mixed as follows: 100 pmol of each crRNA were mixed

with 200 pmol of tracrRNA in 10 mM potassium acetate and 3 mM HEPES (pH 7.5) buffer and incubated at 95°C for 2 min, followed by cooling to room temperature. The annealed crRNA's/tracrRNA complexes were mixed with Cas9 mRNA, Cas9 protein, and *Kcnk2_templ* template DNA. The final concentrations of CRISPR-Cas9 components in 0.6 mM HEPES (pH 7.5) and 2 mM potassium acetate microinjection buffer were as follows: crRNA's (2 pmol/µl) each, tracrRNA (4 pmol/µl), Cas9 mRNA (10 ng/µl), Cas9 protein (25 ng/µl), *Kcnk2_templ* template DNA (20 ng/µl). The final injection solution was filtered through Millipore centrifugal columns and spun at 20,000*g* for 10 min at room temperature. Microinjections were performed in B6D2F1 (hybrid between C57BL/6J and DBA strains) fertilized one-cell oocytes. Oocytes were removed from oviducts of superovulated B6D2F1 female mice in M2 media supplemented with hyaluronidase (400 µg/ml), washed twice for removal of cumulus cells in M2 media, transferred to KSOM media, and kept at 5% CO_2 and 37°C before injection. Cytoplasmic microinjections were performed in M2 media using the Transjector 5246 (Eppendorf), and Narishige NT-88NE micromanipulators attached to a Nikon Diaphot 300 inverted microscope. Oocytes that survived microinjections were transferred to oviducts of pseudopregnant CD1 foster mice and carried to term. Positively targeted F0 and F1 animals were identified by qPCR and sequencing analysis of genomic DNA isolated from tail biopsies.

Kcnk2_floxed mice genotyping: Genotyping of the *Kcnk2_floxed* mice was performed using Kcnk2pcrD12 (TGCTCATCGTCTGATCAAAGTT), LoxA1rev (GCCCTCAAGTAGGTTGTTTCC, and Kcnk2pcrR12 (AAGTGGAGCCCAGGAAGGTTAT) to detect the wild type and floxed alleles. The line was bred to create homozygous $Kcnk2^{fl/fl}$ genotypes.

Animals used for generation of the *Kcnk2_floxed* mouse line: The subjects were males and females of *Kcnk2_flox* mice. All mouse procedures were performed in compliance with the guidelines for

the welfare of experimental animals issued by the Federal Government of Germany. The mouse line was established by breeding male with female C57BL/6J mice to produce heterozygous mice. Pups were weaned at 19 to 23 days after birth, and females were kept separately from males. The mice were housed in standard IVC cages. General health checks were performed regularly in order to ensure that any findings were not the result of deteriorating physical conditions of the animals.

DNA Southern blot analysis: Targeted animals were analyzed using the Southern-blot DNA method. Approximately 5-15 µg of genomic DNA's were digested with restriction endonuclease *Bam*HI or *Pst*I, fractionated on 0.8% agarose gels, and transferred to GeneScreen nylon membranes (NEN DuPont). The membranes were hybridized with a ^{32}Plabeled *Kcnk2_templ* DNA probe containing sequences of the exon 4, loxP sites and intronic flanking regions and washed with (final concentrations) 0.5x SSPE (1x SSPE is 0.18 M NaCl, 10 mM NaH_2PO4, and 1mM EDTA [pH 7.7]) and 0.5% sodium dodecyl sulfate at 65°C.

BaseScope analysis

Endothelial cell-specific deletion of *kcnk2* in *Kcnk2*$^{fl/fl}$*xTie2*^{cre} mice was analyzed using an exon-specific BaseScope™ Duplex in situ hybridization assay (Advanced Cell Diagnostics, ACD), following the manufacturer's instructions. The exon-specific approach was selected because Cre-mediated recombination in the *kcnk2*$^{fl/fl}$ model results in the excision of exon 4, which is critical for gene function. Briefly, 20-µm thick cryosections from brains of *Kcnk2*$^{fl/fl}$*xTie2*$^{cre+}$ and *Kcnk2*$^{fl/fl}$*xTie2*$^{cre-}$ mice immunized for EAE were mounted on Superfrost Plus Gold slides (Menzel) and dehydrated through an ethanol gradient (50%, 70%, and 2× 100%). Sections were treated with hydrogen peroxide for 10 minutes at room temperature, followed by antigen retrieval in 1× RNAscope® Target Retrieval solution (ACD) for 5 minutes at 99°C. Protease III (ACD) treatment was then performed for 30 minutes at 40°C. Hybridization was carried out for 2 hours at 40°C

using probes targeting exon 4 of the *kcnk2* mRNA (probe #440421, ACD) and *cd31* mRNA (probe #852311-C2, ACD) to label endothelial cells. Signal amplification was performed according to the BaseScope™ Duplex reagent kit protocol. Finally, sections were counterstained with 50% Gill's Hematoxylin solution (Sigma-Aldrich) and mounted with Vectamount mounting medium (H-5000; Vector Laboratories).

Implantation of a Carotid Artery Catheter for Drug Administration

Operation procedures for the establishment of a carotid artery catheter were previously published in detail [1]. In brief, 30 min after subcutaneous caprofen administration (4 mg/kg), the mice were anesthetized using 1-2% isoflurane (Abbot) in oxygen and a face mask. The eyes were protected from drying out with dexpanthenol ointment. Mice were turned on the back and fixated with adhesive tape prior to cautious skin incision next to the neck midline. Without detraction of the vagus nerve, the trachea was gently exposed by the separation of fat and muscle and the contiguous carotid artery was ligated using a suture thread followed by artery incision and implantation of the catheter. After this procedure 100 µl of rhodamine B-labeled dextran (Thermo Fisher) was injected intra-arterially for CNS blood vessel visualization. The catheter was blocked with PBS (Gibco) followed by exposure of the brainstem.

Operation and Setup for Two-Photon Excitation Laser Scanning Microscopy

Anesthetized mice were continuously ventilated and were immobilized on a stereotactic frame with the head inclined. For brainstem imaging, we adapted previous imaging protocols [2]. After midline scalp incision, the atlanto-occipital membrane was exposed by cautiously removing the musculature. Next, the dura mater was stripped between the occipital skull bone and the first cervical vertebra. The exposed brainstem was continuously rinsed with PBS using a peristaltic pump. Movement artefacts due to heart pulsation or breathing were reduced to a minimum by the

preparation of a sterile agarose patch (0.5% in 0.9% NaCl solution) on the facing brain. Finally, a region of interest (ROI) containing blood vessels was identified with the laser scanning microscope in the brainstem. To control for a potential influence of time in anesthesia, records were started either without drug administration (baseline) followed by intraarterially injection of 100 µg/kg spadin (Tocris Bioscience) in PBS after 30 min or with spadin injected directly prior to imaging followed by administration of 30 µg αICAM1 antibody [YN1/1.7.4] (BioLegend) via the catheter after 30 min. Imaging location was not affected from drug administration thus providing the same ROI before and after. Dual near-infrared and infrared (IR) excitation of the live brainstem was applied at 850 nm by an automatically tunable Ti:Sa laser (Mai Tai HP, Spectra Physics) and 1,110 nm by an optical parametric oscillator (OPO, APE, Berlin) pumped by the Ti:Sa laser. XYZ-stacks were recorded using a TriMScope I multiphoton system from LaVision Biotec. Volumes of approximately 300 µm x 300 µm x 72 µm were acquired with laser powers ranging from 2-6 mW at the brain surface over time and exported as TIFFs for 3D analyses.

Images were post processed using Imaris software (Bitplane, Switzerland). The cell tracks established by the tracking tool were manually corrected by persons blinded to the experimental setup and treatments. Endoluminar crawling, extravasation of T cells and perivascular crawling were determined manually with 3D rotation and surface analysis.
