# Supplementary material for: The potassium channel K2P2.1 shapes the morphology and function of brain endothelial cells via actin network remodeling
Source: Nat Commun. 2025 Jul 18;16:6622. doi: 10.1038/s41467-025-61816-9 (PMC12274505; doi:10.1038/s41467-025-61816-9)
Supplement: Supplementary file 2 — Description of Additional Supplementary Information [file 41467_2025_61816_MOESM2_ESM.pdf]

Description of Supplementary Material

Supplementary Movie 1 - Intravital two-photon microscope imaging of brainstem of mice.

Representative video of 2PM imaging of the brainstem after EAE induction, at a disease score of 2. Blood vessels were visualized by rhodamine-labelled dextran (red); Th17 T cells are tagged with GFP (green). Baseline recording was performed for 30 min, followed by spadin treatment for K2P2.1 inhibition.
