# Supplementary material for: The potassium channel K2P2.1 shapes the morphology and function of brain endothelial cells via actin network remodeling
Source: Nat Commun. 2025 Jul 18;16:6622. doi: 10.1038/s41467-025-61816-9 (PMC12274505; doi:10.1038/s41467-025-61816-9)
Supplement: Supplementary file 4 — Reporting summary [file 41467_2025_61816_MOESM4_ESM.pdf]

nature portfolio

Corresponding author(s): Ruck, Tobias MD

Last updated by author(s): Jun 18, 2025

Reporting Summary

Nature Portfolio wishes to improve the reproducibility of the work that we publish. This form provides structure for consistency and transparency in reporting. For further information on Nature Portfolio policies, see our Editorial Policies and the Editorial Policy Checklist.

Statistics

For all statistical analyses, confirm that the following items are present in the figure legend, table legend, main text, or Methods section.

n/a	Confirmed	
☐	☒	The exact sample size (n) for each experimental group/condition, given as a discrete number and unit of measurement
☐	☒	A statement on whether measurements were taken from distinct samples or whether the same sample was measured repeatedly
☐	☒	The statistical test(s) used AND whether they are one- or two-sided<br>*Only common tests should be described solely by name; describe more complex techniques in the Methods section.*
☒	☐	A description of all covariates tested
☐	☒	A description of any assumptions or corrections, such as tests of normality and adjustment for multiple comparisons
☐	☒	A full description of the statistical parameters including central tendency (e.g. means) or other basic estimates (e.g. regression coefficient) AND variation (e.g. standard deviation) or associated estimates of uncertainty (e.g. confidence intervals)
☐	☒	For null hypothesis testing, the test statistic (e.g. F, t, r) with confidence intervals, effect sizes, degrees of freedom and P value noted<br>*Give P values as exact values whenever suitable.*
☒	☐	For Bayesian analysis, information on the choice of priors and Markov chain Monte Carlo settings
☒	☐	For hierarchical and complex designs, identification of the appropriate level for tests and full reporting of outcomes
☐	☒	Estimates of effect sizes (e.g. Cohen's d, Pearson's r), indicating how they were calculated

Our web collection on statistics for biologists contains articles on many of the points above.

Software and code

Policy information about availability of computer code

Data collection	Axiovision (Zeiss), Keyence NanoScope software V5.3.1 and V8.10; JPK Cell Hesion(R)200 (Bruker);CytExpert (Beckman Coulter); StepOne Plus and QuantStudio 3 qRT-PCR Platform (Applied Biosystems); DIA-NN V1.7.15; BioRad CFX384
Data analysis	ImageJ (Fiji); PUNIAS 3D; JPK Data Processing software (Bruker); Kaluza Analysis V2.1 (Beckman Coulter); Quiagen GeneGlobe data analysis tool;StepOne Software (Applied Biosystems); Cytoscape V3.8.2; GraphPad Prism V10

For manuscripts utilizing custom algorithms or software that are central to the research but not yet described in published literature, software must be made available to editors and reviewers. We strongly encourage code deposition in a community repository (e.g. GitHub). See the Nature Portfolio guidelines for submitting code & software for further information.

Data

Policy information about availability of data

All manuscripts must include a data availability statement. This statement should provide the following information, where applicable:

- Accession codes, unique identifiers, or web links for publicly available datasets
- A description of any restrictions on data availability
- For clinical datasets or third party data, please ensure that the statement adheres to our policy

Mass spectrometry-based proteomic data have been deposited in the ProteomeXchange Consortium via the PRIDE partner repository with the data set identifier PXD031051.

Research involving human participants, their data, or biological material

Policy information about studies with human participants or human data. See also policy information about sex, gender (identity/presentation), and sexual orientation and race, ethnicity and racism.

Reporting on sex and gender	n.a.
Reporting on race, ethnicity, or other socially relevant groupings	n.a.
Population characteristics	n.a.
Recruitment	n.a.
Ethics oversight	n.a.

Note that full information on the approval of the study protocol must also be provided in the manuscript.

Field-specific reporting

Please select the one below that is the best fit for your research. If you are not sure, read the appropriate sections before making your selection.

☒ Life sciences ☐ Behavioural & social sciences ☐ Ecological, evolutionary & environmental sciences

For a reference copy of the document with all sections, see nature.com/documents/nr-reporting-summary-flat.pdf

Life sciences study design

All studies must disclose on these points even when the disclosure is negative.

Sample size	Sample sizes were calculated based on an expected Cohen's d of > 0.5 (estimated using data of previous comparable studies) to detect medium and large effects witha type I error of α = 0.05 and a power of 0.85 (two-tailed, unpaired t test).
Data exclusions	No data were excluded from analysis.
Replication	All experiments were performed in at least three independent biological replicates to ensure reproducibility of the study.
Randomization	All experiments were conducted under randomized and blinded conditions, both in data acquisition (including scoring of EAE, drug administration) and analysis, and randomization was e.g. done by changing, arbitrary recording and treatment order of the various groups. Primary cells were randomly assigned to the experimental group.
Blinding	Blinding was performed during acquisition and analysis of images, video analysis for cell migration studies and analysis of RT-qPCR.

Reporting for specific materials, systems and methods

We require information from authors about some types of materials, experimental systems and methods used in many studies. Here, indicate whether each material, system or method listed is relevant to your study. If you are not sure if a list item applies to your research, read the appropriate section before selecting a response.

Materials & experimental systems

n/a	Involved in the study	
☐	☒	Antibodies
☒	☐	Eukaryotic cell lines
☒	☐	Palaeontology and archaeology
☐	☒	Animals and other organisms
☒	☐	Clinical data
☒	☐	Dual use research of concern
☒	☐	Plants

Methods

n/a	Involved in the study	
☒	☐	ChIP-seq
☐	☒	Flow cytometry
☒	☐	MRI-based neuroimaging

Antibodies

Antibodies used	rabbit anti-TREK1 (Sigma-Aldrich #T6448, polyclonal); rat anti-ICAM1 (Abcam #25375, clone YN1/1.7.4); Cy2 goat anti-rabbit (Jackson Immuno-Research #111-225-144); Cy3 donkey-anti rat (Dianova # 712-166-153); rabbit anti-Cofilin 1 (Abcam # ab42824); rabbit anti-

	p (S3) Cofilin 1 (Abcam # ab12866); mouse anti-PIP2 (Santa Cruz Biotechnology # sc-53412); AF488 goat anti-mouse (Invitrogen #A28175); purified anti-mouse CD3 antibody (Biolegend #100302, clone 145-2C11); purified anti-mouse CD28 antibody (Biolegend #102102, clone 37.51); BV605 rat anti-mouse CD31 (Biolegend #102427, clone 390), APC/Cy7 rat anti-mouse CD45 (Biolegend #103116, clone 30-F11,) Ultra-LEAF(TM) purified anti-mouse ICAM1 (Biolegend #116132, clone YN/1.7.4)
Validation	rabbit anti-TREK1 (Sigma-Aldrich #T6448, polyclonal): The antibody reacts with rat TREK-1 (confirmed). It is expected that the antibody will also react with human and mouse TREK-1 due to sequence homology; rat anti-ICAM1 (Abcam #25375, clone YN1/1.7.4): reactive to mouse; validated for IHC (PMID: 8943409) and blocking (PMID: 2981916); rabbit anti-Cofilin 1 (Abcam # ab42824): validated by the use in at least 17 publications (PMID: 30824802, 30926595, 30787030, etc.); rabbit anti-p (S3) Cofilin 1 (Abcam # ab12866): validated by the use in at least 6 publications (PMID: 31379509, 30824802, 29115990, etc.); mouse anti-PIP2 (Santa Cruz Biotechnology # sc-53412): validated in at least 27 publications (PMID:30695232, 9425510, 30344928, etc.); purified anti-mouse CD3 antibody (Biolegend #100302, clone 145-2C11): quality tests and validated according to the manufacturer; purified anti-mouse CD28 antibody (Biolegend #102102, clone 37.51): quality tests and validated according to the manufacturer; Ultra-LEAF(TM) purified anti-mouse ICAM1 (Biolegend #116132, clone YN/1.7.4): quality tests and validated according to the manufacturer and publications (PMID:23940206)

Animals and other research organisms

Policy information about studies involving animals; ARRIVE guidelines recommended for reporting animal research, and Sex and Gender in Research

Laboratory animals	Mice: C57BL/6J; Kcnk2-/- ; B6.2D-2.GFP; B6.2D2; Rag2-/-cgn-/-. All mice were used at the age of 8-15 weeks; EAE was performed with female mice, exclusively, for primary cell isolation both, male and female mice were used.
Wild animals	No wild animals were used in this study.
Reporting on sex	See above: both male and female mice were used.
Field-collected samples	No field-collected samples were used in this study.
Ethics oversight	The animal work within this study was approved by the local institutional Animal Care Committee and appropriate state committees for animal welfare (AZ: A17.019; G14-1-038)

Note that full information on the approval of the study protocol must also be provided in the manuscript.

Plants

Seed stocks	n.a.
Novel plant genotypes	n.a.
Authentication	n.a.

Flow Cytometry

Plots

Confirm that:

☒ The axis labels state the marker and fluorochrome used (e.g. CD4-FITC).

☒ The axis scales are clearly visible. Include numbers along axes only for bottom left plot of group (a 'group' is an analysis of identical markers).

☐ All plots are contour plots with outliers or pseudocolor plots.

☒ A numerical value for number of cells or percentage (with statistics) is provided.

Methodology

Sample preparation	Primary isolated MBMECs were used directly after isolation. Cells were stained for CD31,CD45,ICAM1,VCAM and K2P2.1. CD31+CD45- cells were identified as endothelial cells and used for further analysis of expression levels of ICAM1, VCAM1 and K2P2.1.
Instrument	Data were acquired using the CytoFlex S (Beckman Coulter).

Software	Data acquisition was visualized using the CytExpert software (Beckman Coulter). Analysis was performed with Kaluza Analysis V2.1 (Beckman Coulter).
Cell population abundance	In this study no sorting and subsequent purity check was performed. Only qualitative statements, but no quantitative conclusions about expression levels were drawn.
Gating strategy	Positive and negative populations were defined based on unstained reference samples and Fluorescent Minus One (FMO) controls in multicolor experiments.

☐ Tick this box to confirm that a figure exemplifying the gating strategy is provided in the Supplementary Information.
